# Does receiving a cash grant improve individual earnings in a war-torn country?  Evidence from a randomized experiment in Afghanistan

**DOI:** 10.12688/f1000research.72893.1

**Published:** 2021-11-15

**Authors:** Fatema Kashefi, Hisahiro Naito

**Affiliations:** 1 Ministry of Labor and Social Affairs, Government of the Islamic Republic of Afghanistan, Kabul, Afghanistan; 2 Graduate School of Humanities and Social Sciences, University of Tsukuba, Tsukuba, Ibaraki, 305-8571, Japan

**Keywords:** Cash Grant; Income; Poverty; RCT; Afghanistan;microenterprise

## Abstract

Background: In this study, the effect of receiving a non-trivial cash grant and mentorship for business practice on individual earnings in Afghanistan was assessed. Methods: This randomized control trial (RCT) included 2177 individuals (n=2177), with the age range of 18–35 years. The amount of cash grant (500 USD) was approximately equal to the gross domestic product (GDP) per capita in the country. By a process of lottery, eligible applicants were equally divided into the group that received the grant (treatment group) and the group that did not (control group). Results: Almost two years after this study, the percentage of increased annual income to the size of this cash grant was approximately 173 % and 69 % for males and females, respectively. This result was much higher than the estimated percentage reported in previous literature assessing the effect of microfinance loans and cash grants in other countries. The treatment group had 7% higher probability of having an officially registered business than the control group. The treatment group also increased labor supply and employed additional workers, compared to the control group. The male treatment group bought more capital equipment (e.g., trike and commercial carts) than the male control group, while the female treatment group bought more domestic animals. Conclusion: A non-trivial cash grant has a strong positive effect on the earnings of the recipient of the cash grant in Afghanistan. If the state of increased income continues for the next few years, the sum of increased income will be more than the sum of the administration cost, the interest cost and the amount of cash grant given. This implies that the project passes the standard criteria to determine the appropriateness of government program. Additionally, we did not find any economically and statistically significant positive effect of mentorship on income.

## Introduction

The Lack of adequately paying jobs has become a major challenge in Afghanistan. In 2016–2017, according to the Afghanistan Living Conditions Survey, 25% of the population were unemployed and 54% lived below the poverty line ( Islamic Republic of Afghanistan Central Statistics Organization, 2018). Even among those employed, their employment is mainly informal and through microenterprises. World bank (2019) reports that 80% of those employed have unstable jobs by either being self-or family employed or having short-term (daily) employment in informal microenterprises.

However, the prevalence of informal microenterprise is not unique to Afghanistan but common among the majority of developing countries ( La Porta & Shleifer, 2014). Thus, the main question would be how the prevalence of informal microenterprises can be transformed into an opportunity for income growth and stable employment.

In the last decade, through randomized experiments, a growing number of studies have indicated the strong and positive effect of cash grants on income growth and microenterprise activities in developing countries ( Blattman *et al.*, 2014; Blattman *et al.*, 2020; De Mel *et al.*, 2008; De Mel *et al.*, 2012; Fafchamps *et al.*, 2014; Haushofer & Shapiro, 2016; McKenzie, 2017). In Afghanistan, however, there are several reasons as to why such cash grants might not be effective in increasing microenterprise activities and income. First, the lack of security is still a major issue in this country. According to the United Nations Assistance Mission to Afghanistan, the number of civilian death due to conflicts and explosives was over 3000 yearly, from 2014 till 2017( United Nations Assistance Mission in Afghanistan, 2018). Second, the female unemployment rate (41%) is higher than the male unemployment (18%), which is mainly due to the negative attitude and lack of social acceptance and support for women in business ( Allen *et al.*, 2007; Beath *et al.*, 2013). Third, Afghanistan is recognized as one of the most corrupted countries worldwide. ( United Nations Office on Drugs and Crime, 2012). In the corruption perception index, which ranks 180 countries and territories based on their recognized levels of public sector corruption, Afghanistan was ranked at fourth from the bottom in 2017 ( Transparency International, 2017). Several official documents report that the corruption in Afghanistan hinders the effectiveness of aid programs from enhancing enterprise activities ( Special Inspector General for Afghanistan Reconstruction, 2018; Special Inspector General for Afghanistan Reconstruction, 2021). As such if weak microenterprise activities are mainly due to these three factors, then providing cash grants in Afghanistan might not be as effective as in other countries.

In this study, we have analyzed the effects of receiving a non-trivial amount of cash grant in Afghanistan for individuals aged 18–35 on their income growth.

Our study has several unique aspects. First, most previous studies with the same topic were conducted in relatively peaceful countries, except for post-conflict studies in Uganda by Blattman *et al.* (2014) and Blattman *et al.* (2020). However, Afghanistan has been at war for the past 20 years, which ended recently ( Wall Street Journal, 2021). Thus, knowledge on how to improve people's income in such a country will be useful for other fragile war-torn countries that need reconstruction. Second, Afghanistan is an Islamic country where Islam with a strong norm, is practiced. In the strictest interpretation of the religion, lending money with interest is prohibited ( Nagaoka, 2012). Therefore, it is important to evaluate whether receiving a cash grant, which does not involve the repayment of the principal and interest, would be effective in this country. Third, in this study, we provide a large amount of cash grant, which is almost equal to the gross domestic product (GDP) per capita. Fourth, most importantly, Islamic Republic of Afghanistan ended on August 15, 2021, without much resistance to Taliban ( Wall Street Journal, 2021). The fact that Taliban took over the country quite easily may indicate that people had already lost trust in the government even before 2021, because the aid programs were not working well due to bad designs and implementation of those programs ( BBC, 2021; Special Inspector General for Afghanistan Reconstruction, 2018). Thus, it is important to evaluate to what degree is this specific government program effective.

## Methods

### Study environment

High population growth rate of Afghanistan along with the lack of security in the country creates a challenging environment for reasonable job opportunities. Weak security conditions make it difficult to conduct business, while reducing the incentive for new businesses. 

According to Afghanistan Living Conditions Survey 2016–2017 ( Islamic Republic of Afghanistan Central Statistics Organization, 2018), unemployment rate defined as the ratio of people who are seeking a job in the labor force but do not have one, was 25% in this country. In particular, the male unemployment rate was 18%, whereas the female unemployment rate was 41%, in that year. Even among those who were employed, 19.7% of males and 23.7% of females stated that they were seeking additional work. Among those employed, 17.2% were salaried employees (including public sector). Specifically, unpaid male family employees were 24.5%, and daily labor employees were 15.7%. It was reported that 40.1% were business owners.

In 2017, during this experiment, the Special Inspector General for Afghanistan Reconstruction reported that approximately 59.7% of the country's 407 districts were under Afghanistan government control or influence; 11.1% of total districts were under insurgent control or influence; 29.2% of all districts were contested districts. ( Special Inspector General for Afghanistan Reconstruction, 2017). According to the United Nations Assistance Mission to Afghanistan, the number of civilian death due to conflicts and explosives was 3701, 3565, 3510, and 3428, in 2014, 2015, 2016, and 2017, respectively ( United Nations Assistance Mission in Afghanistan, 2018). Meanwhile, the number of injuries caused by these activities for the same years was 6384, 7419, 7924, and 7015, respectively.

 In terms of education, there is a substantial disparity between males and females, depending on the area. For example, in urban areas, 66.9% of males and 40.8% of females are literate, whereas in rural areas these values decrease to 45.6% and 13.1% for males and females, respectively. The poverty rate is also severe in Afghanistan. In 2016, it was reported that 54% of households had expenditure less than the poverty threshold.

###  Study design

The program "Promoting Entrepreneurship among the Youth in Afghanistan" (PEYA) was a Randomized Control Trial (RCT)-based project funded by the Afghan Reconstruction Trust Fund. It was launched in August 2015 and ended in July 2018. The PEYA program was implemented by the Ministry of Labor and Social Affairs of Afghanistan in Balkh, Kabul, and Nangarhar provinces. The program's objective was to help establish new microenterprises or expand existing businesses by providing a cash grant of USD 500 (approximately 33,500 Afghani (Afs)), which is approximately equivalent to 2017 GDP per capita. The amount of this grant can be considered significant in a country with high levels of unemployment and illiteracy. In this study, 3490 eligible applicants were chosen depending on their business proposals and social economic background. Based on a lottery process, half of the study participants (1745) were assigned to the treatment group where they received a cash grant, whilst the other half who were assigned to the control group did not. The lottery and the baseline interview were conducted in August 2016. Due to the potential lack of sufficient numbers of business mentors in all provinces, mentorships were randomly given to half of the individuals who received the cash grant and lived in Kabul.

As presented in Table 1, the key eligibility criteria for the applicants were male and female aged between 18–35 years, who were illiterate or had a minimum education, and who resided in one of three provinces: Balkh, Kabul, and Nangarhar. The selected applicants had to have a national ID (Tazkira) and a bank account. This project was implemented through the collaboration of four teams: i) the Non-formal Approach to Training, Education, and Jobs in Afghanistan team (NATEJA), ii) the Provincial Directories of the Ministry of Labor and Social Affairs, iii) the Employment Service Centers (ESCs) (set up for better coordination of the intervention), and iv) the implementing team of the Ministry of Labor and Social Affairs.

**Table 1.** Summary of the Intervention (Baseline).

	Description
Grant Size	USD 500 or AFN 33500 [AFN might change slightly due to exchange rate]
Key Eligibility	Age 18–35 and illiterate (slight Flexibility, minimum education would be considered.)
Coverage Area	3 provinces: Kabul, Balkh and Nangarhar
Target Market	Mainly micro and few small enterprises
Distribution Quota	Male 70%, Female 30%

Source:Narrative Progress Report (1st Quarter: Jan to Mar 2016) for subcomponent 3.2 (Promoting Entrepreneurship among Afghan Youth) August 8 2016, Non-formal approach to training education and jobs in Afghanistan(NATEJA)

**Figure 1.**
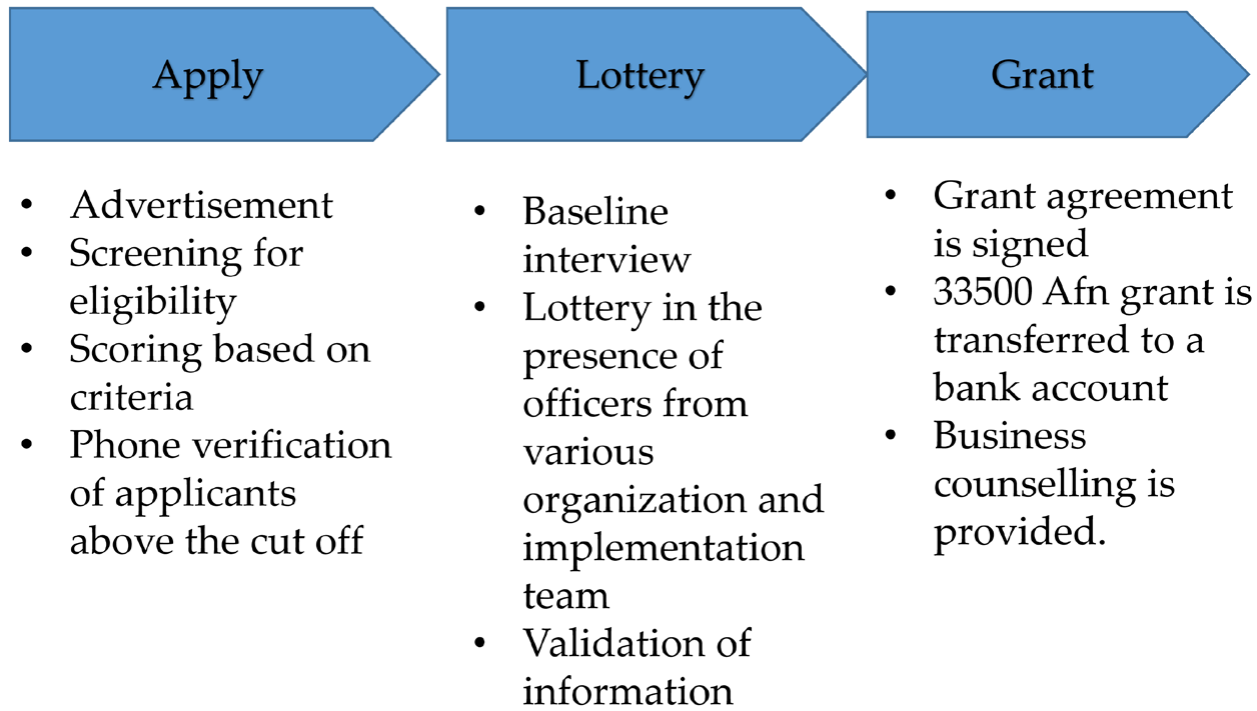
The procedure of the Experiment. Source:Narrative Progress Report (1st Quarter: Jan to Mar 2016) for subcomponent 3.2 (Promoting Entrepreneurship among Afghan Youth) August 8 2016, Non-formal approach to training education and jobs in Afghanistan(NATEJA).

Figure 1 shows the procedure of the experiment. The selection process of the grantees started by providing information about the intervention and eligibility criteria. Four districts in each province were specified. The operation teams were assigned to mobilize the communities in these areas. This process lasted for 30 days, and each community was visited twice. During the initial briefing (week 1) and refresher briefing (week 2), information about the objective and selection criteria was also shared with local authorities, such as the governor's office staff, provincial councils, and community leaders, to have their support.

The application form was a three-page questioner containing 19 questions that covered six sections (skills, support, and investment; product/services; price; place; promotion; customers). The answers to most questions were closed-ended; however, there were several questions that had open-ended options to provide space for detailed answers. Each question and option had equivalent marks. Completion of the questioner on average took 15–20 minutes. Quality assurance was made by the training and moderating committee at the local level. Collectedly, the project had 8238 applications from the three provinces ( Non-formal approach to training education and jobs in Afghanistan(NATEJA), 2016).

Considering the low level of literacy within the communities, application forms printed on large banners were also provided in addition to conventional brochures and posters. Subsequently, the applicants were selected based on an evaluation of their initial submitted business plan, which included information, such as education, age, demographic characteristics, income, work experience, business type, and their plans on how they would spend the grant. In many cases, the applicants were assisted by other family members, neighbors, etc., who had a higher level of education and were able to convey the information in the application form. The applications with a higher score than the threshold level of the score become qualified for the lottery process. After the qualified applicants were selected, scheduled meetings for baseline interviews and lottery were announced to be held in each province. At the meetings, professionally trained interviewers conducted the baseline interviews. After which each qualified applicant drew a lottery number that determined the receipt of the cash grant.

Given the amount of the cash grant and concern about corruption in the process of choosing the recipients, it was important that all applicants understood that the recipients of cash grants were chosen in a transparent manner. Thus, the process of lottery took place in the presence of representatives from provincial councils, provincial governors, the Implementing Partner Agency (IPA), ESCs, and representatives of provincial directorates of labor and social affairs. After determining the recipients of the grant, a three-member panel verified the information provided, and the grant agreement was signed. Finally, the full grant was transferred in one transaction to the grantees' submitted bank account.

At the endline, the evaluation teams started to interview individuals among those who participated at the baseline, from June 2018 till July 2018. During this period a total of 3005 individuals were interviewed. However, due to miscommunication between the Ministry of Labor and Social Affairs and the external teams the study ID numbers and national ID numbers for some surveyed individuals in the endline that could match their endline with the baseline data, was not recorded by the external teams. To ensure that correct matching was implemented, we included only individuals whose study ID number and national ID number were matched with the baseline and the endline data in the final analysis sample. As a result, the final sample size was 2177 individuals (n=2177) in this study, with the number of the individuals in the treatment group (1146) slightly higher than that of the control group (1031) at the endline ( Table 2), implying 38 % attrition rate.

**Table 2.** The Number of Observations in Baseline and Endline.

Survey	(1) All	(2) Control	(3) Treatment
Baseline data set (August 2016) (percent of the sample)	3490	1745 50%	1745 50%
Endline data set (June-July 2018) (percent of the sample)	3005	1340 44.59%	1665 55.41%
Cleaned Data set (percent of the sample)	2177	1031 47.38%	1146 52.62%

Notes: For the endline data, due to administrative erros, the matching id that matches the endline data with the baseline data were lost due to miscommunication between the ministry of labor and social affairs and the data collecting company. As a result, the sample size of the cleaned data set, which matches the baseline data set with the endline data set is much smaller than the endline data set surveyed.

In order to address the potential bias that can occur due to the high attrition rate, several robust bounding methods have been used in this study ( Lee, 2009; Manski, 1990) to control this high attrition rate, as presented in the robustness checks.

### Statical model

As the PEYA program is a RCT experiment, we estimate the effect of the policy by estimating the intent to treat (ITT) with the following analysis of covariates specification:


$$Y_{i,A}={\text{β}}_{0}+{\text{β}}_{1}\;Grant_{i}+{\text{β}}_{2}Y_{i,B}+{\text{α}}_{1}X_{i,B}+{\text{ε}}_{i},\;(1)$$

where i is the index of an individual, *Y _i,A_* is the outcome variable in the endline period (e.g., monthly earnings in the last month) for individual i, *Y _i,B_* is the outcome variable at the baseline of individual i, and Grant *_i_* is the dummy variable which is equal to one if individual i received the grant and zero if not.

As discussed, the grant was randomized at each individual level. Thus, to calculate the standard error, we used the robust standard error. *β*_1_ captures the impact of the cash grant; *X _i,B_* is a set of control variables at baseline, which includes age group dummy, province dummy, years of schooling, and zero education dummy; and *ε _i_* refers to the individual error term. We estimated the above equation using ordinary least squares (OLS). In order to address the potential bias that can occur due to the high attrition rate, several robust bounding methods were applied in this study ( Lee, 2009; Manski, 1990). Stata version 16 was used for the study analysis.

## Results

### Balance test at baseline

Table 3 shows the summary statistics of baseline variables and the balancing test of the sample used in the study analysis. Panel A shows that of the 2177 participants, 72% of the individuals were male, and 42% were in 18–23 age group. Moreover, 54% of the participants were married at baseline. Approximately 47% of them were heads of the household at baseline.

**Table 3.** Summary Statistics of Baseline Variables and Balancing Tests.

	(1) No Grant	(2) Receiving Grant	(3) Difference	(4) p-value
Variables	Mean	Mean	(1)-(2)	
A. Demographic Variables				
Years of schooling	2.501	2.588	-0.087	0.440
No Education	0.445	0.426	0.019	0.363
Male	0.724	0.712	0.012	0.551
Married	0.555	0.531	0.023	0.274
Household size	7.474	7.727	-0.253 **	0.049 **
Head of Household	0.462	0.470	-0.009	0.687
Aged 18–23	0.427	0.471	-0.044 **	0.037 **
Aged 24–29	0.308	0.281	0.027	0.161
Aged 30–35	0.265	0.248	0.017	0.366
B. Region and Native Language				
Balkh	0.317	0.340	-0.023	0.251
Kabul	0.372	0.352	0.021	0.314
Nangarhar	0.310	0.308	0.002	0.906
Pashu Speaker	0.465	0.456	0.008	0.701
Dari/Persian Speaker	0.502	0.513	-0.011	0.619
Other Language	0.032	0.029	0.003	0.664
C. Income, Business and Assets Related Variables				
New to business	0.919	0.922	-0.004	0.743
Monthly Earning in Afs.	4015.2	3925.1	90.2	0.606
No earning in last month	0.339	0.341	-0.003	0.895
Main Occupation is agricultrual	0.017	0.022	-0.004	0.463
Number of plots	0.581	0.717	-0.136	0.224
Own the House	0.725	0.754	-0.029	0.119
Main Occupation is non- agricultrual	0.983	0.978	0.004	0.463
Livestock and animals	0.325	0.344	-0.019	0.351
p-value of joint significance test				0.3326
N	1031	1146		

Notes. The total sample size is 2177. The sample size of the male sample is 1562 and the and sample size of the female sample is 615. All variables are from the baseline survey. The joint significant test regresses the treatment dummy on baseline covariates and then testing the estimated coefficients are all equal to zero. For regresses the treatment dummy on baseline covariates, we eliminated age 30-35 dummy, Nanghar dummy, and other langue dummy due to multi-colinearity. *** p<0.01, ** p<0.05, * p<0.1.

Panel B shows the geographic distribution of the three provinces and their native language. The geographic distribution is evenly apportioned between the treatment and control groups. This panel shows that approximately 51% of the participants have stated Dari language as their mother tongue, 46% of them were Pashto native speakers, and 3% were minority language speakers (i.e., Turkemni, Uzbeki, and Pashai).

Panel C shows income-related information at the baseline. The results indicate that 92% of the individuals in our study were new to business, implying that they had not setup their businesses at the baseline. Specifically, the average income in the last month at the baseline was approximately 4,000 Afs. The P-value (0.33) of the joint significant test as shown in Table 3, shows that our analysis sample was balanced.

**Figure 2.**
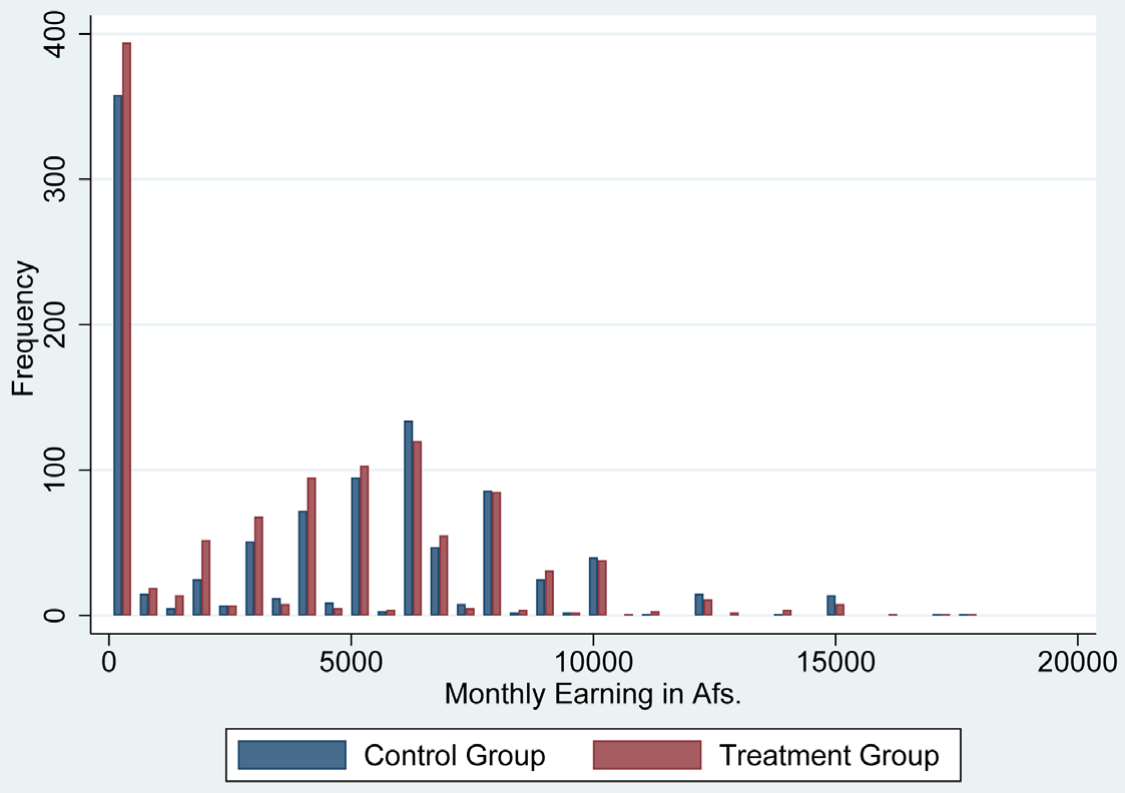
The monthly income at baseline.

Figure 2 shows the histogram of monthly income for those who had received cash grants and those who had not at baseline. According to estimates by the National Statistical Office in a 2016–2017 study, the poverty line threshold was 2,064 Afs per person per month ( Islamic Republic of Afghanistan Central Statistics Organization, 2018). Thus, the average income in our study sample was two times higher than the income at the poverty line. However, as shown in Figure 2, a substantial percentage of the population had zero income. Figure 2 also shows that the distribution of income was quite similar between those who received cash grants and those who did not.

**Figure 3.**
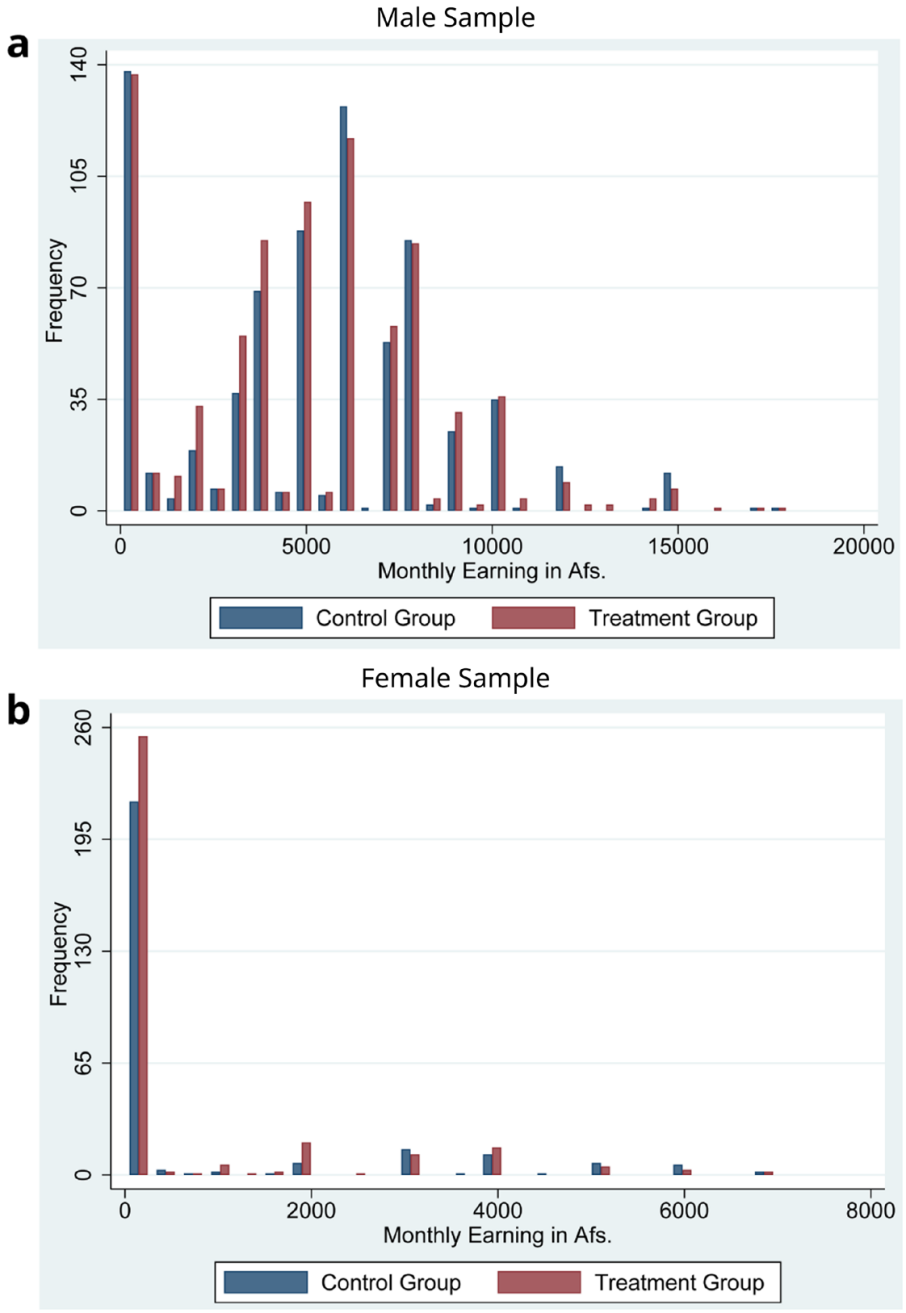
The monthly income of the male and female groups at baseline.

Figure 3a and 3b display the histogram of monthly earnings for the male and the female groups, respectively. Figure 3a and 3b show that the distributions of earnings for the male and the female samples were quite different. For example, less than 20% of the male sample have zero earnings. However, more than 70 % of the female sample have zero earnings.

Table 4 shows the mean and balancing test of the key variables of the baseline variables in the male group. About 42% of this group were without education, and 57% were married. The average monthly income for this group was 5130 Afs, although 17% were without income.

**Table 4.** Summary Statistics of Baseline Variables and Balancing Tests (Cash Grant) for Male Sample.

	(1) No Grant	(2) Receiving Grant	(3) Difference	(4) p-value
Variables	Mean	Mean	(1)-(2)	
A. Demographic Variables				
Years of schooling	2.484	2.613	-0.129	0.325
No Education	0.436	0.413	0.023	0.366
Married	0.571	0.572	-0.001	0.960
Household size	7.540	7.767	-0.227	0.148
Head of Household	0.601	0.609	-0.009	0.731
Aged 18–23	0.394	0.434	-0.040	0.111
Aged 24–29	0.320	0.304	0.016	0.484
Aged 30–35	0.286	0.262	0.023	0.304
B. Region and Native Language				
Balkh	0.340	0.360	-0.020	0.413
Kabul	0.369	0.354	0.014	0.553
Nangarhar	0.291	0.286	0.005	0.816
Pashu Speaker	0.507	0.501	0.005	0.829
Dari/Persian Speaker	0.458	0.464	-0.006	0.812
Other Language	0.035	0.033	0.002	0.848
C. Income, Business and Assets Related Variables				
New to business	0.928	0.918	0.010	0.472
Monthly Earning in Afs.	5126.9	5141.7	-14.9	0.939
No earning in last month	0.177	0.167	0.010	0.591
Main Occupation is agricultrual	0.024	0.029	-0.005	0.518
Number of plots	0.730	0.916	-0.186	0.228
Own the House	0.753	0.783	-0.030	0.518
Main Occupation is non-agricultrual	0.976	0.971	0.005	0.187
Livestock and animals	0.330	0.362	-0.032	0.187
p-value of joint significance test				0.9
N	746	816		

Notes. The total sample size of the male sample is 1562. The joint significant test regresses the treatment dummy on baseline covariates and then testing the estimated coefficients are all equal to zero. For regressing the treatment dummy on baseline covariates, we drop age 30–35 dummy Namghar dummy and other langue dummy due to multi-collinearity. *** p<0.01, ** p<0.05, * p<0.1C.

Table 5 shows the mean and balancing test of the key variables in the female group. About 47% of this group were without education, and 48% were married. The average monthly earnings of this group were 5130 Afs; however, 76 % were without income.

**Table 5.** Summary Statistics of Baseline Variables and Balancing Tests (Cash Grant) for Female Sample.

	(1) No Grant	(2) Receiving Grant	(3) Difference	(4) p-value
Variables	Mean	Mean	(1)-(2)	
A. Demographic Variables				
Years of schooling	2.547	2.527	0.020	0.927
No Education	0.470	0.458	0.013	0.755
Married	0.512	0.430	0.082 **	0.042 **
Household size	7.302	7.627	-0.326	0.136
Head of Household	0.098	0.127	-0.029	0.255
Aged 18–23	0.512	0.564	-0.051	0.203
Aged 24–29	0.277	0.224	0.053	0.132
Aged 30–35	0.211	0.212	-0.002	0.962
B. Region and Native Language				
Balkh (Dummy)	0.256	0.291	-0.035	0.335
Kabul (Dummy)	0.382	0.345	0.037	0.343
Nangarhar (Dummy)	0.361	0.364	-0.002	0.954
Pashu Speaker	0.354	0.345	0.009	0.817
Dari/Persian Speaker	0.618	0.633	-0.016	0.687
Other Language	0.025	0.018	0.006	0.588
C. Income, Business and Assets Related Variables				
New to Business	0.895	0.933	-0.039 *	0.091 *
Monthly Earning in Afs.	1105.3	916.5	188.7	0.418
No earning in last month	0.761	0.773	-0.011	0.741
Main Occupation is agricultrual	0.000	0.003	-0.003	0.318
Number of plots	0.189	0.224	-0.035	0.458
Own the House	0.649	0.682	-0.033	0.318
Main Occupation is non-agricultrual	1.000	0.997	0.003	0.742
Livestock and animals	0.312	0.300	0.012	0.742
p-value of joint significance test				0.315
N	285	330		

Notes. The total sample size of the female sample is 615. The joint significant test is regressing the treatment dummy on baseline covariates and then testing the estimated coefficients are all equal to zero. For regressing the treatment dummy on baseline covariates, we drop age 30–35 dummy, Nanghar dummy and other langue dummy. *** p<0.01, ** p<0.05, * p<0.1.

Table 6 displays the balancing test regarding the mentorship program. The p-value of the joint significant test shows that the data is balanced between the group who received the mentorship and who did not.

**Table 6.** Summary Statistics of Baseline Variables and Balancing Tests (Mentorship).

	(1) No Mentorship	(2) Mentorship	(3) Difference	(4) p-value
Variables	Mean	Mean	(1)-(2)	
A. Demographic Variables				
Years of schooling	2.501	2.588	-0.066	0.440
No Education	0.445	0.426	-0.013	0.363
Male	0.724	0.712	0.018	0.551
Married	0.555	0.531	0.092 *	0.274
Household size	7.474	7.727	0.043	0.049 **
Head of Household	0.462	0.470	0.077	0.687
Aged 18–23	0.427	0.471	-0.067	0.037 **
Aged 24–29	0.308	0.281	0.006	0.161
Aged 30–35	0.265	0.248	0.061	0.366
B. Native Language				
Pashu Speaker	0.383	0.406	-0.023	0.640
Dari/Persian	0.612	0.589	0.023	0.641
Other Language	0.000	0.005	-0.005	0.318
C. Income, Business and Assets Related Variables				
New to business	0.871	0.866	0.004	0.898
Monthly Earning in Afs.	3810.95	3252.2	558.72	0.232
No earning in last month	0.363	0.396	-0.033	0.498
Main Occupation is agricultrual	0.035	0.025	0.010	0.553
Number of plots	0.363	0.356	0.007	0.903
Own the House	0.637	0.668	-0.032	0.508
Main Occupation is non-agricultrual	0.965	0.975	-0.010	0.553
Livestock and animals	0.194	0.223	-0.029	0.479
p-value of joint significance test				0.9426
N	201	202		

Notes. The sample is individuals who received the grant and lived in Kabul. The joint significant test is regressing the mentorship dummy on baseline covariates and then testing the estimated coefficients are all equal to zero. For regressing the treatment dummy on baseline covariates, we eliminated age 30–35 dummy Nanghar dummy and other langue dummy. *** p<0.01, ** p<0.05, * p<0.1.

Table 7 displays the index of different types of assets between the control and treatment groups. These assets were divided into Group 1: productive durable, including animal ownership, and local transportation equipment (e.g., cart, three-wheel/truck, and bike), and Group 2: consumer durable, including household goods (e.g., home appliances and mobile phones. To construct household asset-related indices, the values of each variable were standardized to ensure that the value of each variable represents its difference from the mean. Then, the variables are compiled into indices using factor analysis.

**Table 7.** Summary Statistics of Assets at Baseline and Balancing Tests.

	(1) No Grant	(2) Receiving Grant	(3) Difference	(4) p-value
Variables	Mean	Mean	(1)-(2)	
Number of plots	-0.027	0.024	-0.052	0.230
Bicycles	-0.012	0.011	-0.023	0.592
Carts	0.006	-0.005	0.011	0.800
Motor bikes	0.009	-0.008	0.017	0.687
Cars	0.031	-0.028	0.059	0.169
Trucks/3wheels	0.004	-0.004	0.008	0.851
Tractors	0.013	-0.011	0.024	0.573
Simple mobiles	-0.013	0.012	-0.025	0.562
Simple mobiles of the respondent	-0.019	0.017	-0.037	0.391
Smart phones/android	0.018	-0.016	0.035	0.421
Smart phones/android of the respondent	0.012	-0.011	0.023	0.587
Radio	-0.026	0.023	-0.049	0.253
TVs	0.018	-0.017	0.035	0.414
Dish Antennas/Satellite	0.007	-0.006	0.012	0.773
Fridges	-0.026	0.024	-0.050	0.246
Computers	-0.023	0.021	-0.044	0.311
Computers with internet access	-0.010	0.009	-0.018	0.672
Cows	0.016	-0.014	0.030	0.489
Oxen/buffalos	-0.009	0.008	-0.017	0.693
Sheep	-0.002	0.001	-0.003	0.941
Goats	-0.035	0.032	-0.067	0.121
Donkeys	0.021	-0.019	0.040	0.349
Horses	-0.014	0.013	-0.027	0.532
Chickens/Turkeys	0.006	-0.005	0.011	0.792
p-value of the joint significant test				0.49
Household Assest for Production Index	-0.004	0.004	-0.009	0.843
Household Assest for Consumption Index	-0.011	0.010	-0.021	0.625
Household Assest Index	-0.012	0.011	-0.023	0.588
N	1031	1146		

Notes: The level of assets is standardardized including the baseline and the endline. Thus, many values are negative in the baseline. *** p<0.01, ** p<0.05, * p<0.1

In our analysis sample, according to Table 7, the p-value of the joint significant test was 0.49, which shows that the mean was balanced between the control and treatment groups.

Despite the high attrition rate, overall, the balancing tests shown in Table 3 – Table 7 show that the balance between the treatment and control groups was maintained in the analysis sample.

### The effect of receiving cash grant and mentorship

Table 8, column 1 shows the effectiveness of receiving cash grants on monthly earnings of the entire group. These results show that receiving cash grants increased the treatment group's monthly earnings by approximately 4000 Afs. As such, the increased annual income will be 48,000 Afs, which is more than the amount of the grant that an individual in the treatment group received. Considering the 33,500 Afs cash grant, the percentage of the increased annual income to the amount of the cash grant would therefore be:

**Table 8.** The Effect of Receiving Cash Grant on Monthly Earnings.

Dependent Variable	(1)	(2)	(3)	(4)	(5)	(6)
	Monthly Earnings in Afs. (Endline)					
Sample Variable	All	Male	Female	All	Male	Female
Cash Grant	3,950 *** (567.5)	4,839 *** (768.6)	1,939 *** (289.7)	4,155 *** (593.4)	5,005 *** (820.5)	2,075 *** (335.8)
Mentorship				-394.3 (944.7)	-225.9 (1,302)	-918.0 (607.6)
Control Variables				Yes	Yes	Yes
N	2,177	1,562	615	2,177	1,562	615
R-squared	0.020	0.023	0.064	0.105	0.080	0.125

Notes. Robust standard errors in parentheses. OLS estimation is applied. Control variables are, the receipt of mentorship dummy, monthly earnings at the baseline, years of schooling, no education dummy, gender dummy (full sample), marital status, household size, head of household dummy, age group dummies, province dummies, language dummies, new to business dummies, zero earnings dummy, number of plots, the house ownership dummy, non-agricultural business dummy, having animal livestock dummy. All control variables are from the baseline information. The estimated coefficients of control variables are shown in Table S1 of Extended Data *** p<0.01, ** p<0.05, * p<0.1.

(48000/33500) × 100 = 143%.

Column 2 displays the effect of receiving a cash grant on monthly earnings in the male group. This shows that receiving the cash grant increased the monthly earnings by about 4839 Afs, and therefore the percentage of the increased annual income to the amount of the cash grant for this group was 173%.

Column 3 displays the effect of receiving the grant on monthly income on the female group. The column shows that receiving the cash grant increased the monthly earnings by about 1900 Afs, which is less than half of the estimated effect on the male group. This implies that the percentage of the increased annual income to the amount of the cash grant for this group was 69%.

Thus, in either of these three cases, if the state of increased income continues for the next few years, the sum of increased income will be more than the sum of the administration cost, the interest cost and the amount of cash grant given. This implies that the project passes the standard criteria to determine the appropriateness of government program.

In columns 4–6, we added several important covariates and checked whether our estimated coefficients were sensitive to the inclusion of those control variables. These columns indicate that the estimated coefficients of the grant variable reported in columns 1–3 are not sensitive to the inclusion of various control variables ( Extended data) ( Naito, 2021b). Additionally, the results in Table 8 show that providing mentorship did not influence the rise in the monthly earnings.

As mentioned previously, the endline study sample size was much smaller than that of the baseline due to the administrative error during the data collection. One concern was whether such attrition of the sample affects the estimated coefficients. Therefore, we controlled for the attrition by applying the inverse probability weighted (IPW) estimation ( Wooldridge, 2002; Wooldridge, 2010) ( Table 9, panel A). To calculate the standard error correctly, we use the Generalized Moment Method estimation (GMM) with IPW.

**Table 9.** Robustness Checks by Controlling Attrition. The Effect of Receiving Cash Grant on Monthly Income.

Dependent Variable	(1)	(2)	(3)
	Monthly Earnings in Afs. (Endline)		
Sample	All	Male	Female
A. Inverse Probability Weighted Estimation with Sample Selection			
Cash Grant	3,984 *** (589.6)	4,876 *** (798.4)	2,062 *** (327.3)
Mentorship	Yes	Yes	Yes
Control Variables	Yes	Yes	Yes
N	3,490	2,450	1,040
B. Bounds based on Imputation			
Cash Grant	1,366 *** (394.7)	2,016 *** (551.5)	1,230 *** (213.5)
Mentorship	Yes	Yes	Yes
Control Variables	Yes	Yes	Yes
N	3,490	2,450	1,040
C. Lee Bounds			
Lower bound	2,137 * (1,186)	2,328 * (1,407)	1,637 * (934.0)
Upper bound	4,891 *** (1,168)	5,761 *** (1,233)	2,614 *** (679.1)
N	3,490	2,450	1,040

Notes: Robust standard errors in parentheses. Control variables are the same as the ones used in Table 8. *** p<0.01, ** p<0.05, * p<0.1.

Table 9, column 1 of Panel A shows the estimation results of applying IPW to the full sample. It shows that the effect of receiving the cash grant on monthly earnings was 3984 Afs, which was quite similar to the estimated coefficient in column 4 of Table 8 (i.e., 4036 Afs). Column 2 displays the estimated coefficient using IPW for the male group. The estimated coefficient was 4876 Afs, similar to the estimated coefficient displayed in column 5 of Table 8 (i.e., 4984 Afs). Column 3 of Panel A presents the estimated coefficient for the female group by using IPW. The estimated coefficient is 2062, which is close to the estimated coefficient in column 6 of Table 8 (i.e., 2070 Afs). Panel A shows that, when attrition is controlled by IPW estimation, the estimated coefficients of the cash grant were similar to the estimated coefficients obtained in the OLS estimation presented in Table 8.

Table 9, Panel B shows bounding treatment effects. As demonstrated by Karlan & Valdivia (2011); Manski (1990), and Blattman *et al.* (2014), we first imputed the outcome variable for the unfound sample by assuming that the monthly earnings of unfound treatment group members were equal to the average monthly earning of the found treatment group minus 0.25 standard deviation of the monthly earnings of the found members. We also assumed that the monthly earnings of the unfound control group were the average monthly earnings of the found control group plus 0.25 standard deviation of the found members. After this imputation, we estimated the effects of the cash grant and other covariates using both the found and unfound samples. With this assumption, we found that receiving the cash grant increased the monthly income by 2000 Afs and 1200 Afs for the male and female groups, respectively. Thus, the cash grant is likely to have increased the monthly earnings.

To further correct for attrition bias, Lee's treatment effect bounds for receiving the cash grant on monthly earnings was estimated ( Table 9, Panel C) ( Lee, 2009). The lower bound of the effect of receiving the cash grant was 2328 Afs and 1637 Afs for the male and female groups, respectively. The OLS estimates were between the lower and upper bounds for each specification and the lower bounds, 2328 Afs and 1637 Afs for the male and female groups were statistically different from zero (p<0.01). Thus, even with conservative estimates, the percentage of the increased annual income to the amount of the cash grant would be 83% for the male group and 58% for the female group.

Overall, analysis based on several estimation methods demonstrates that the effect of receiving the cash grant on monthly earnings was economically and statistically significant.

Table 10 shows the effect of the cash grant on having an officially registered business, labor supply and asset levels. In this table, Panel A displays the effect of the cash grant on the probability of having an officially registered business at endline. Column 1 of Panel A shows that an individual who received the cash grant had an 8% higher probability of having an officially registered business on average. Column 2 and 3 show that male and female individuals who received the cash grant have 7% and 9% higher probability of having an officially registered business, respectively. As shown in columns 4–6, we added several important control variables. The columns also show that the estimated coefficients of the treatment dummy were similar to the estimated coefficient in columns 1–3.

**Table 10.** Effect of Cash Grant on Business, Employment and Asset Levels.

Sample	(1) All	(2) Male	(3) Female	(4) All	(5) Male	(6) Female
Panel A.						
Dependent Variable	Having an Officially Registered Business (Endline)					
Cash Grant	0.0769 *** (0.0169)	0.0702 *** (0.0206)	0.0943 *** (0.0284)	0.0768 *** (0.0180)	0.0695 *** (0.0220)	0.0972 *** (0.0313)
Mentorship				0.00435 (0.0369)	0.00925 (0.0445)	-0.00760 (0.0674)
R-squared	0.012	0.012	0.018	0.034	0.036	0.054
Panel B.						
Dependent Variable	Number of Household Members Working for the Enterprise (Endline)					
Cash Grant	0.201 *** (0.0278)	0.180 *** (0.0311)	0.254 *** (0.0582)	0.194 *** (0.0308)	0.191 *** (0.0356)	0.193 *** (0.0600)
Mentorship				0.0113 (0.0726)	-0.0640 (0.0639)	0.185 (0.193)
R-squared	0.022	0.020	0.028	0.040	0.039	0.091
Panel C.						
Dependent Variable	Number of Non-household Members Working for the Enterprise (Endline)					
Cash Grant	0.232 *** (0.0519)	0.232 *** (0.0504)	0.229 * (0.132)	0.224 *** (0.0551)	0.248 *** (0.0533)	0.202 (0.131)
Mentorship				0.0450 (0.0913)	-0.0242 (0.0993)	0.175 (0.198)
R-squared	0.009	0.013	0.005	0.037	0.062	0.039
Panel D.						
Dependent Variable	Index of Household Assets for Production (Endline)					
Cash Grant	0.142 *** (0.0427)	0.176 *** (0.0558)	0.0626 (0.0522)	0.143 *** (0.0425)	0.167 *** (0.0555)	0.0735 (0.0490)
Mentorship				-0.0763 (0.0578)	-0.0778 (0.0744)	-0.0923 (0.0810)
R-squared	0.005	0.006	0.003	0.166	0.182	0.056
Panel E.						
Dependent Variable	Index of Household Assets for Consumption (Endline)					
Cash Grant	0.0686 (0.0426)	0.100 ** (0.0490)	-0.0210 (0.0840)	0.0509 (0.0408)	0.0935 * (0.0481)	-0.0722 (0.0779)
Mentorship				0.0300 (0.0613)	0.00544 (0.0688)	0.0843 (0.128)
R-squared	0.001	0.003	0.000	0.254	0.232	0.331
Sample Size and Inclusion of Control Variables						
Control Variables				Yes	Yes	Yes
N	2,177	1,562	615	2,177	1,562	615

Notes: Robust standard errors in parentheses. OLS estimation is applied. Control variables are, the receipt of mentorship dummy, the outcome variable at the baseline, years of schooling, no education dummy, gender dummy (full sample), marital status, household size, head of household dummy, age group dummies, province dummies, language dummies, new to business dummies, zero earnings dummy, number of plots, the house ownership dummy, non-agricultural business dummy, having animal livestock dummy. All control variables are from the baseline information. The estimated coefficients of control variables are shown in Table S2-S5 of Extended Data *** p<0.01, ** p<0.05, * p<0.1

 Panel B shows the effect of the cash grant on the number of household members working for a microenterprise. Panel B shows that an individual who received the cash grant increased the number of household members working for their microenterprise by 0.2 persons on average. Column 2 of Panel A, a male individual who received the cash grant increased the number of family members working for their microenterprise by 0.18 persons on average. Column 3 shows that a female individual who received the cash grant increased the number of family members working for their micoenterprise by 0.254 persons on average.

Panel C shows the effect of the cash grant on the number of non-household members working for a microenterprise. Column 1 shows an individual who received the cash grant increased the number of non-family members who work for their microenterprise by 0.23 persons. Column 2 shows that, for the male group who had received the cash grant, the employment number of non-family members increased by 0.232 persons. The results in column 3 show that, for the female group who had received the cash grant, the number of non-family members who were employed for their microenterprise increased by 0.229 persons.

When starting or extending a business, additional equipment, such as phone, truck, and cart, might be needed. Panel D shows the effect of receiving cash grants on the general asset index for production. To calculate the general asset index for production, we first standardized the level of each asset for production. Then, through the principal component analysis, we determined the weight for each asset and calculated the weighted index ( Jolliffe, 2005). As shown in columns 2 and 3, we found that, receiving the cash grant increased standard deviations of the index of assets for production by 0.176 and 0.062 for the male and the female group, respectively. When the control variables were included, the cash grant increased the index of assets for production by 0.167 standard deviation for the male group and by 0.074 standard deviation for the female group (columns 5 and 6).

Results in Panel E display the index of assets for consumption. Columns 2 and 3 show that receiving the cash grant increased the index of assets for consumption by 0.1 standard deviation for the male group, while decreasing it by 0.02 standard deviation for the female group. When the control variables were included in the estimation, the estimated coefficient and statistical significance did not change. Columns 5 and 6 show that the cash grant increased the index of assets for consumption for the male group by 0.094 standard deviation, while decreasing it by 0.073 standard deviation for the female group. Thus, for the male group, receiving the cash grant increased the index of assets for consumption. However, our results do not reflect the same for the female group.

Table 11 shows the effect of receiving the cash grant on the levels of different types of assets. The results in this table show that, for the male group, receiving the cash grant increased the level of assets, e.g., bicycles, carts, three-wheel trucks, mobile phones, and cows. Conversely, the number of cows was the only asset that increased after receiving the cash grant in the female group.

**Table 11.** Effect of Cash Grant on Level of Different Types of Assets at Endline.

Sample	(1) All	(2) Male	(3) Female	(4) All	(5) Male	(6) Female
Panel A.						
Dependent Variable	z-score of Bicycle (Endline)					
Cash Grant	0.0758 * (0.0425)	0.0822 * (0.0460)	0.0600 (0.0939)	0.0522 (0.0449)	0.0542 (0.0472)	0.0587 (0.112)
Panel B.						
Dependent Variable	z-score of Cart (Endline)					
Cash Grant	0.164 *** (0.0425)	0.181 *** (0.0480)	0.129 (0.0863)	0.144 *** (0.0449)	0.143 *** (0.0500)	0.142 (0.0912)
Panel C.						
Dependent Variable	z-score Three Wheel Truck (Endline)					
Cash Grant	0.0506 (0.0430)	0.0971 *** (0.0373)	-0.0672 (0.120)	0.0462 (0.0470)	0.0949 ** (0.0418)	-0.0275 (0.122)
Panel D.						
Dependent Variable	z-score Simple Moblie Phone (Endline)					
Cash Grant	0.148 *** (0.0424)	0.175 *** (0.0489)	0.0782 (0.0846)	0.150 *** (0.0431)	0.170 *** (0.0509)	0.0917 (0.0792)
Panel E.						
Dependent Variable	z-score Refrigerator (Endline)					
Cash Grant	0.0958 ** (0.0421)	0.139 *** (0.0521)	-0.0167 (0.0689)	0.0835 ** (0.0416)	0.133 ** (0.0525)	-0.0423 (0.0653)
Panel F.						
Dependent Variable	z-score of Cows (Endline)					
Cash Grant	0.428 *** (0.0410)	0.426 *** (0.0514)	0.439 *** (0.0626)	0.409 *** (0.0415)	0.389 *** (0.0521)	0.450 *** (0.0633)
Control Varialbes				Yes	Yes	Yes
N	2,177	1,562	615	2,177	1,562	615

Notes. Robust standard errors in parentheses. OLS estimation is applied. Control varialbles are, the receipt of mentorship dummy, the outcome variable at the baseline, years of schooling, no education dummy, gender dummy (full sample), matrial status, household size, head of household dummy, age group dummies, province dummies, language dummies, new to business dummies, zero earnings dummy, number of plots, the house ownership dummy, non-agricultural business dummy, having animal livestock dummy.All control variables are from the baseline information.*** p<0.01, ** p<0.05, * p<0.1.

## Discussion

The government of Islamic Republic of Afghanistan collapsed on August 15, 2021 ( Wall Street Journal, 2021). The minimal resistance to Taliban takeover, may indicate that all the efforts by the western societies for development of this country might not have been entirely effective ( BBC, 2021). Several official documents by the western governments state that development aids to enhance enterprise activities in Afghanistan failed due to bad implementation of the programs ( Special Inspector General for Afghanistan Reconstruction (2018); Special Inspector General for Afghanistan Reconstruction (2021)).

In contrast, the results of this study have shown that the effect of a non-trivial cash grant on income is substantial in Afghanistan. The percentage of increased annual income to the size of this cash grant almost two years after receipt of the cash grant was approximately 173% for males and 69% for females. The treatment group had a 7% higher probability of having an officially registered business than the control group. The treatment group also increased labor supply and employed additional workers, compared to the control group. The male treatment group bought more capital equipment (e.g., trike and commercial carts) than the male control group, while the female treatment group bought more domestic animals. Our results are consistent with the previous studies in other countries which similarly show a positive effect of cash grants on income ( Blattman *et al.*, 2014; Blattman *et al.*, 2020; De Mel *et al.*, 2008, De Mel *et al.*, 2012; Fafchamps *et al.*, 2014; Haushofer & Shapiro, 2016; McKenzie, 2017). However, our estimated coefficient is higher than the estimated coefficients in the previous literature.

We would like to emphasize, however, that this study had some limitations. First, the endline survey was conducted almost two years after the study. Thus, the short-term effect of cash grants was measured. Blattman *et al.* (2020) who studied the long-term effects of grants on poverty, have shown that cash grants are ineffective after 9 years. Thus, it is possible that the strong effect of the cash grant seen in this study might be less significant in the long term. Second, this PEYA program focuses on microenterprises, not small- or medium-sized enterprises. Those two factors might explain the difference between our results and the assessment of several official documents of the aid programs in Afghanistan. Third, as emphasized in the introduction, Afghanistan is a country with fragile security; thus, the strong effect might be due to this situation. For example, it is possible that, due to the security situation of the country, many existing businesses were destroyed. As a result, when individuals start businesses with cash grants, there might not be so much competition in the existing markets. If this is the reason for the high return from the cash grants, the applicability of our results can be limited to countries whose security situation is very weak.

 As discussed in the introduction, initially there was a concern that giving cash grants may not enhance economic activities in Afghanistan due to negative perception toward women in business and the prevalence of corruption. First, our estimation results show that although the effect of the cash grant on female individuals was lower than the effect on their male counterparts, the effect on earnings of female individuals was strong enough to justify the cash grant. Second, despite the prevalence of corruption in Afghanistan, the effect of the cash grant was strong. One possible reason for the effectiveness of this program might be due to the transparency of the selection of the recipients of the cash grants. As such, the project paid special attention to the lottery process to ensure random selection of these individuals. This implies that although the lottery process prevents the project from selecting the best candidates, it also prevents a case in which the project chooses corrupted and incapable individuals, a case which often happens in the government contracts in Afghanistan ( Special Inspector General for Afghanistan Reconstruction, 2018; Special Inspector General for Afghanistan Reconstruction, 2021). This may indicate that random selection might be effective in countries with high corruption rates.

 Third, in this study, randomization for the receipt of the cash grant was done at individual level instead of village level. This implies that if there is a positive/negative externality of the cash grant, and if treatment and control individuals tend to live in the same village, the effect of the cash grant is underestimated/overestimated. In our context, a positive externality is likely if the recipient of the cash grant starts a new business, employs other individuals, and increases the income of other individuals as well as his/her own income. A negative externality is likely if the recipient of the cash grant starts a new business in an existing market and decreases the profit of the control individuals. In an extended study ( Extended data) ( Naito, 2021b), Global Positioning System (GPS) information was also used to determine whether the distance of each individual to the nearest treatment individual affects the estimated coefficient of the cash grant dummy. We found that there is no systematic correlation between the distance and the estimated size of the coefficient of the cash grant dummy. This suggests that it is unlikely that the experimental design that uses individual level randomization induces a systematic bias.

 According to the International Monetary Fund (IMF), the lending interest rate in Afghanistan in 2016 was 15% ( International Monetary Fund, 2018). Given the high percentage of increased income to the amount of the cash grant and the relatively low lending interest rate, a question arises as to why individuals do not borrow money for business investments. Plausible explanations could be capital constraints and/or limited risk-sharing. Finding the correct mechanism is important because policy implications depend on the correct mechanism, as different mechanisms imply different policy implications. For example, if the lack of a risk-sharing mechanism is the source of the strong effect of the cash grant, then a policy that enhances the income risk-sharing of the business activities, such as progressive taxation on income with subsidy on business investment, is likely to enhance the welfare. If the underlying mechanism is the inaccessibility to capital, then a policy that makes capital more available such as public lending, will improve welfare. Future studies are needed to examine the underlying mechanisms.

## Conclusions

Non-trivial cash grants had a substantial effect on the monthly earnings of the treatment group two years after this study. The percentage of increased annual income to the size of this cash grant two years after the experiment is approximately 173% for males and 69% for females. We did not find evidence that receiving mentorship on business increases monthly earnings.

It is still not clear as to why receiving a large amount of cash grant has such a significant effect on the monthly earnings. To investigate, further longitudinal studies are needed to not only confirm the results of this study but to utilise quantitative and qualitative strategies to assess how such a large cash grants can affect the economy of a household.

## Data availability

### Underlying data

Open Science Framework (OSF): The effect of receiving cash grant on income in Afghanistan, https://doi.org/10.17605/OSF.IO/JQHRP ( Naito, 2021a)

This project contains the following underlying data:

- Questionnaire_cash_grant_afghanistan.zip. survey questionnaires
- Summary_stat1.do: stata file for summary statistics
- F1000_regression.do: stata file for regression analysis
- F1000_sample_selection_model.do: stata file for generalized moment method with inverse probability weighted estimation and bounding estimation

The above data are available under the terms of the Creative Commons Zero "No rights reserved" data waiver ( CC0 1.0 Public domain dedication)

The RCT dataset used in this study is owned by the World Bank Group. This RCT dataset can be requested from the World bank group ( infoafghanistan@worldbank.org).

The findings, interpretations, and conclusions expressed in this article are entirely those of the authors and should not be attributed in any manner to the World Bank, to its affiliated organizations, or to members of its Board of Executive Directors, or the countries they represent. The World Bank does not guarantee the accuracy of the data included in this publication and accepts no responsibility for any consequence.

### Extended data

Open Science Framework (OSF): The effect of receiving cash grant on income in Afghanistan: Extended Data, https://doi.org/10.17605/OSF.IO/QG49F ( Naito, 2021b).

This project contains the following extended data:

- Extended_data.pdf: additional regression results

Data are available under the terms of the creative commons zero "no rights reserved" data waiver ( CC0 1.0 Public domain dedication) 

### Reporting guidelines

Open Science Framework (OSF): CONSORT checklist for 'The effect of receiving cash grant on income in Afghanistan', https://doi.org/10.17605/OSF.IO/JQHRP ( Naito, 2021a).

Data are available under the terms of the Creative Commons Zero "No rights reserved" data waiver ( CC0 1.0 Public domain dedication)
