# Supplementary material for: Does receiving a cash grant improve individual earnings in a war-torn country?  Evidence from a randomized experiment in Afghanistan
Source: F1000Res. 2023 Apr 12;10:1156. Originally published 2021 Nov 15. [Version 2] doi: 10.12688/f1000research.72893.2 (PMC10576184; doi:10.12688/f1000research.72893.2)
Supplement: Peer review history

# Reviewer report: Reviewer response for version 2

**DOI:** 10.5256/f1000research.145450.r169354

**Published:** 2023-10-13

**Authors:** Svetlana Vlady

**Affiliations:** 1 Department of Accounting, Brooklyn College, City University of New York, New York, NY, USA

Authors added a more comprehensive literature review on the impact of cash grants and provided recommendations.

 The research is interesting and timely; it relates in a coherent and cogent way to issues of real-world significance. The authors evaluated the effect of implementing a government program and conducted research on how the prevalence of informal microenterprises can be transformed into an opportunity for income and employment growth.

 In Afghanistan, where a quarter of the population is unemployed and more than half of the population lives below the poverty line, the government implemented a program that helped establish new microenterprises by providing a cash grant. The authors make an original contribution to knowledge by investigating the effect of cash grants on income growth in the context of Afghanistan.

 The uniqueness of this research is that it focuses on a country that suffers from a lack of security, a high level of corruption, a low level of literacy, and gender inequalities. Afghanistan is an Islamic country with rigid social norms and a high female unemployment rate due to a negative attitude toward women empowerment and a lack of support for women in business. Nevertheless, research found that cash grant has a strong positive effect on the earnings of the recipients. However, this research found that the increase in annual income was 2.5 times higher for males than females.

 The article demonstrates the applicability, relevance, and effectiveness of the program and will be useful for other fragile or war-torn countries that need reconstruction. Also, receiving a cash grant, which does not involve the repayment of principal and interest, would be effective in Islamic countries.

 Conclusions are clear and insightful, as they are drawn from the data,

Is the work clearly and accurately presented and does it cite the current literature?

Yes

If applicable, is the statistical analysis and its interpretation appropriate?

I cannot comment. A qualified statistician is required.

Are all the source data underlying the results available to ensure full reproducibility?

No source data required

Is the study design appropriate and is the work technically sound?

Yes

Are the conclusions drawn adequately supported by the results?

Yes

Are sufficient details of methods and analysis provided to allow replication by others?

Yes

Reviewer Expertise:

NA

I confirm that I have read this submission and believe that I have an appropriate level of expertise to confirm that it is of an acceptable scientific standard.

# Reviewer report: Reviewer response for version 1

**DOI:** 10.5256/f1000research.76502.r137413

**Published:** 2022-06-20

**Authors:** Svetlana Vlady

**Affiliations:** 1 Department of Accounting, Brooklyn College, City University of New York, New York, NY, USA

The research is interesting and timely; it relates in a coherent and cogent way to issues of real-world significance. The authors evaluated the effect of implementing a government program and conducted research on how the prevalence of informal microenterprises can be transformed into an opportunity for income and employment growth.

 In Afghanistan, where a quarter of the population is unemployed and more than half of the population lives below the poverty line, the government implemented a program that helped establish new microenterprises by providing a cash grant. The authors make an original contribution to knowledge by investigating the effect of cash grants on income growth in the context of Afghanistan.

 The uniqueness of this research is that it focuses on a country that suffers from a lack of security, a high level of corruption, a low level of literacy, and gender inequalities. Afghanistan is an Islamic country with rigid social norms and a high female unemployment rate due to a negative attitude toward women empowerment and a lack of support for women in business. Nevertheless, research found that cash grant has a strong positive effect on the earnings of the recipients. However, this research found that the increase in annual income was 2.5 times higher for males than females.

 The article could be enriched by a literature review of previous research on the effect of cash grants in other countries and a discussion on culture-specific differences in gender roles at home and in the business in Afghanistan.

 The article demonstrates the applicability, relevance, and effectiveness of the program and will be useful for other fragile or war-torn countries that need reconstruction. Also, receiving a cash grant, which does not involve the repayment of principal and interest, would be effective in Islamic countries.

 Conclusions are clear and insightful, as they are drawn from the data, but could be expanded by recommendations. Minor proofreading and check results by qualified statisticians are suggested.

Is the work clearly and accurately presented and does it cite the current literature?

Yes

If applicable, is the statistical analysis and its interpretation appropriate?

I cannot comment. A qualified statistician is required.

Are all the source data underlying the results available to ensure full reproducibility?

No source data required

Is the study design appropriate and is the work technically sound?

Yes

Are the conclusions drawn adequately supported by the results?

Yes

Are sufficient details of methods and analysis provided to allow replication by others?

Yes

Reviewer Expertise:

Cross-cultural differences, education, oil and gas industry, sustainabilty.

I confirm that I have read this submission and believe that I have an appropriate level of expertise to confirm that it is of an acceptable scientific standard, however I have significant reservations, as outlined above.

# Response

**Published:** 2023-03-10

**Authors:** Hisahiro Naito

**Affiliations:** University of Tsukuba, Japan

Reply to the second referee

 Thank you for reading our manuscript and providing constructive comments.

**Comment 1:** “ *The article could be enriched by a literature review of previous research on the effect of cash grants in other countries and a discussion on culture-specific differences in gender roles at home and in the business in Afghanistan.*”

**Reply: **On page 3, we added a section regarding the literature on the effect of cash grants and provided a comprehensive literature survey. Furthermore, on page 2, we discuss culture-specific differences in gender roles at home and in business in Afghanistan. We hope that our literature survey and discussion on the role of culture in women’s lives are satisfactory.

**Comment 2: **” *Conclusions are clear and insightful, as they are drawn from the data, but could be expanded by recommendations. Minor proofreading and check results by qualified statisticians are suggested.*”

**Reply**: We provide some recommendations in the Conclusion section. More specifically, we wrote, “The results could indicate that the cash grant is a useful tool to increase income in a country that suffers from a lack of security, a high level of corruption, a low level of literacy, and gender inequalities.” We also asked a professional company to proofread our revised manuscript. The research field of one of the authors is applied econometrics. In the past, Naito conducted theoretical research on econometrics and published a paper. He checked the econometric aspects of this paper thoroughly.

# Reviewer report: Reviewer response for version 1

**DOI:** 10.5256/f1000research.76502.r121770

**Published:** 2022-03-01

**Authors:** Ellina Samantroy Jena

**Affiliations:** 1 V.V. Giri National Labour Institute, New Delhi, India

The paper is based on an interesting issue pertaining to economic empowerment through micro enterprises. The gender inequalities in terms of access to  income was apparent in case of women. The paper would have benefitted more from literature highlighting the gendered constraints of women .The paper has highlighted that the percentage of annual income to the size of cash grant was greater for males in comparison to females. The reason for this could have provided a fresh insight into the study, for example women's access to resources, finances *etc.* and the challenges they face. 

 The paper definitely raises certain questions that need more in depth research in future. On the whole, the paper is based on a very important topic and may be considered for indexing after checking the statistical part from trained statisticians.

 As the authors have clearly indicated that further studies on qualitative aspects are required. I would suggest to explore the qualitative aspects with a focus on gender dimensions particularly contextualizing women in fragile contexts may be in another paper in future.

Is the work clearly and accurately presented and does it cite the current literature?

Yes

If applicable, is the statistical analysis and its interpretation appropriate?

I cannot comment. A qualified statistician is required.

Are all the source data underlying the results available to ensure full reproducibility?

No source data required

Is the study design appropriate and is the work technically sound?

Yes

Are the conclusions drawn adequately supported by the results?

Yes

Are sufficient details of methods and analysis provided to allow replication by others?

Yes

Reviewer Expertise:

gender and labour market research, unpaid work,time use studies, gender ,labour laws and international labour standards

I confirm that I have read this submission and believe that I have an appropriate level of expertise to confirm that it is of an acceptable scientific standard.

# Response

**Published:** 2023-03-10

**Authors:** Hisahiro Naito

**Affiliations:** University of Tsukuba, Japan

Reply to the first referee

 Thank you for reading our manuscript, approving it, and giving constructive comments. As you suggested, in our future research, we are planning to explore the points you suggested, that is, the qualitative aspect of gender in Afghanistan.

 Thank you.
